# High-resolution human KIR genotyping

**DOI:** 10.1007/s00251-021-01247-0

**Published:** 2022-01-20

**Authors:** Jonathan Downing, Lloyd D’Orsogna

**Affiliations:** 1 Department of Clinical Immunology, PathWest, Perth, WA Australia; 2 School of Biomedical Sciences, University of Western Australia, Perth, WA Australia

**Keywords:** Killer-cell immunoglobulin receptor (KIR), Next-generation sequencing (NGS), Allele genotyping

## Abstract

Killer immunoglobulin-like receptors (KIR) regulate the function of natural killer cells through interactions with various ligands on the surface of cells, thereby determining whether natural killer (NK) cells are to be activated or inhibited from killing the cell being interrogated. The genes encoding these proteins display extensive variation through variable gene content, copy number and allele polymorphism. The combination of KIR genes and their ligands is implicated in various clinical settings including haematopoietic stem cell and solid organ transplant and infectious disease progression. The determination of KIR genes has been used as a factor in the selection of optimal stem cell donors with haplotype variations in recipient and donor giving differential clinical outcomes. Methods to determine KIR genes have primarily involved ascertaining the presence or absence of genes in an individual. With the more recent introduction of massively parallel clonal next-generation sequencing and single molecule very long read length third-generation sequencing, high-resolution determination of KIR alleles has become feasible. Determining the extent and functional impact of allele variation has the potential to lead to further optimisation of clinical outcomes as well as a deeper understanding of the functional properties of the receptors and their interactions with ligands. This review summarizes recently published high-resolution KIR genotyping methods and considers the various advantages and disadvantages of the approaches taken. In addition the application of allele level genotyping in the setting of transplantation and infectious disease control is discussed.

## Introduction: KIR genes and their receptors

### KIR structure and function

**Fig. 1:**
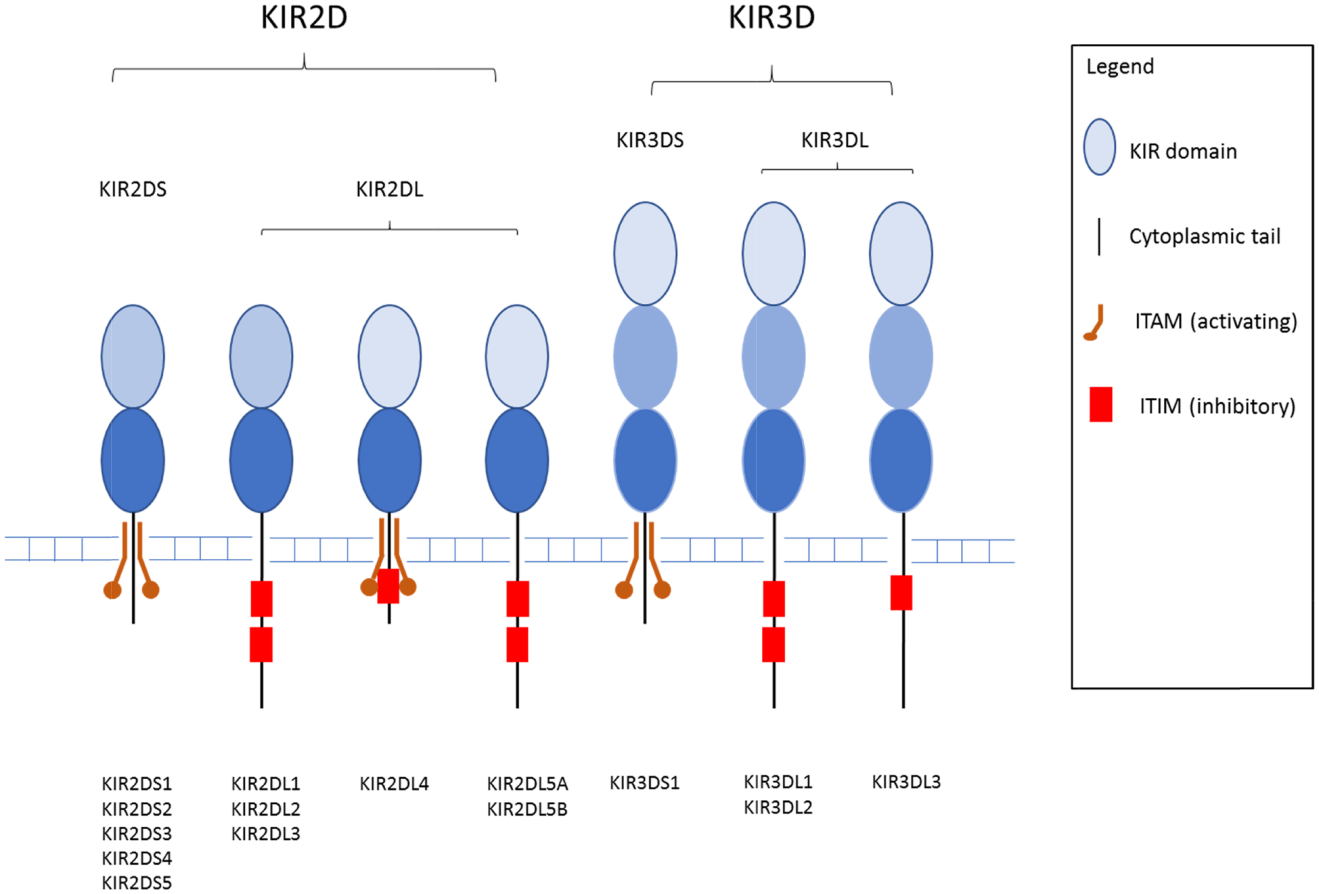
Structural organisation of KIR proteins

The killer immunoglobulin-like receptor (KIR) genes, located on chromosome 19, encode a family of cell surface-expressed transmembrane proteins that regulate the killing of virus infected or malignant cells by natural killer (NK) cells and some T cell subsets (Wende 1999). So far, 17 genes have been described which broadly fit into two groups: inhibitory or activating, depending on whether the protein has a long (L) [2DL1, 2DL2, 2DL3, 2DL4, 2DL5A, 2DL5B, 3DL1, 3DL2, 3DL3] or short (S) [2DS1, 2DS2, 2DS3, 2DS4, 2DS5, 3DS1] cytoplasmic tail. In addition, there are two pseudogenes [2DP1, 3DP1]. Figure 1 shows a schematic of KIR protein structural organisation. The inhibitory KIR possess an immunoreceptor tyrosine–based inhibitory motif (ITIM) within their cytoplasmic domain (Blery et al. 1997), whereas activating KIR bring about activation via crosslinking with DAP12 a disulphide-bonded homodimer containing an immunoreceptor tyrosine–based activation motif (ITAM) (Lanier et al. 1998). The extracellular Ig domains are designated D_0_, D_1_ and D_2_ moving from distal to proximal to the cell membrane. The KIR3D receptors possess the D_0_, D_1_ and D_2_ domains; KIR2DL4 and 2DL5 possess D_0_-D_2_; all other KIR2D receptors possess D_1_ and D_2_. The ligands for inhibitory KIR are known to be autologous HLA-class I molecules and therefore help maintain tolerance to healthy self-tissues.

**Fig. 2:**
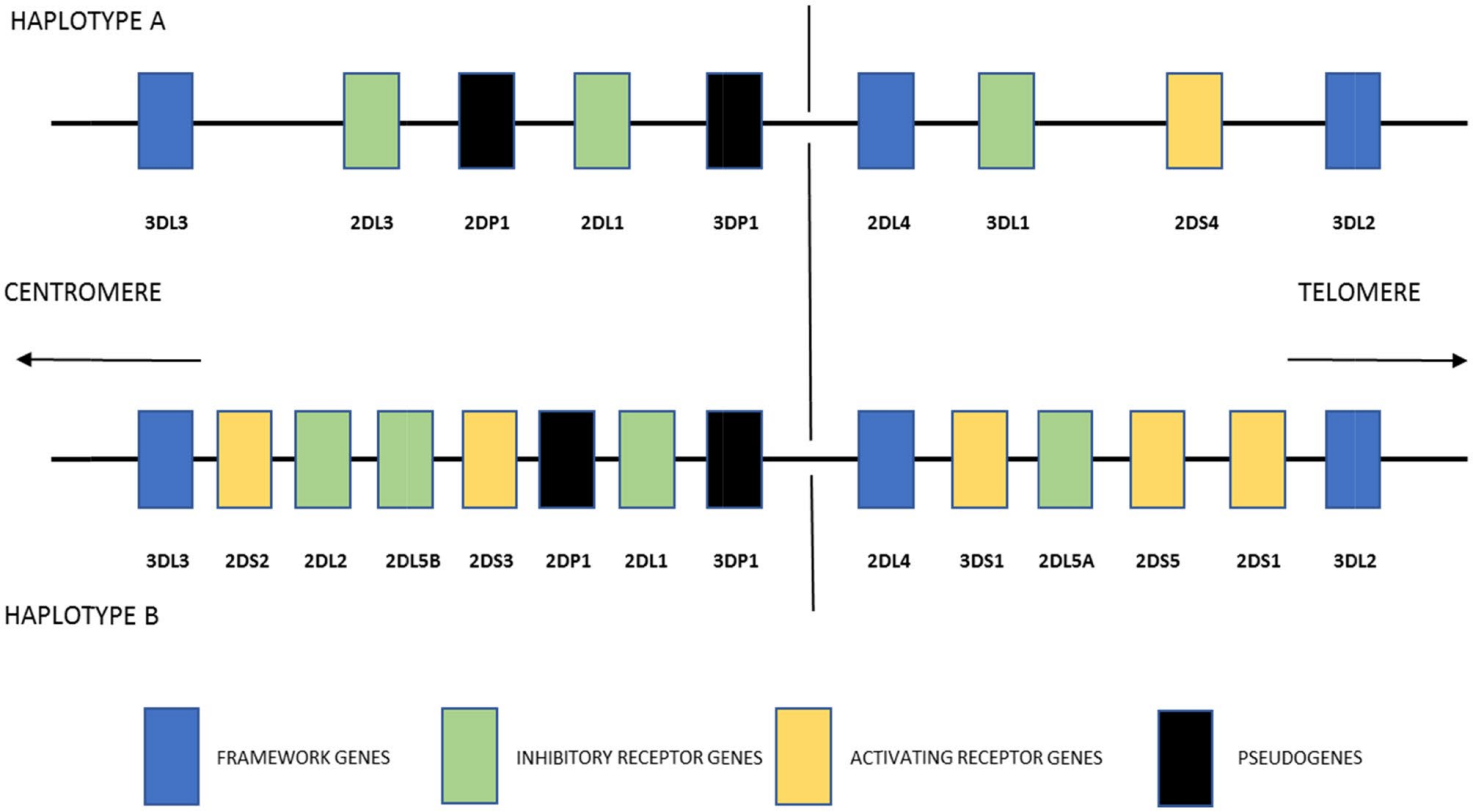
Haplotype gene content

Historically, KIR genes have mostly been ‘typed’ to determine their presence or absence within the individual’s genome. The KIR genes show a high degree of variation in individual gene content and gene copy number, but are also highly organized. The genes are located in the leucocyte receptor complex (LRC) between LILR and FCAR and sit head to tail with regular spacing. Framework genes KIR3DP1, KIR2DL4, KIR3DL2 and KIR3DL3 occur in nearly every individual and flank regions of variability (Wilson et al. 2000). At the haplotype level, KIR haplotypes are classified into two groups, namely A and B. Figure 2 shows the gene content of the A and B haplotypes. The KIR A haplotype has been defined as containing KIR3DL3, 2DL3, 2DL1, 2DL4, 3DL1, 2DS4 and 3DL2; whereas the B haplotype shows more variation and has more genes for activating receptors (Uhrberg et al. 1997). An early haplotype model theorized that the centromeric portion of KIR haplotypes segregated into five different types, whereas the telomeric portion segregated into two different types producing ten different ‘prototypic’ haplotypes, with further variation within these (Hsu et al. 2002). This model sees the centromeric portion as bound by the KIR3DL3 and KIR3DP1 framework genes and can possess KIR2DS2, KIR2DL2 or KIR2DL3, KIR2DL5, KIR2DS3 or KIR2DS5, KIR2DP1 and KIR2DL1. Following KIR3DP1 towards the telomeric end, framework KIR2DL4 delineates the telomeric portion which is then bound by the final framework gene KIR3DL2. This portion can then contain KIR3DL1 with either KIR2DS1 or KIR2DS4; or KIR3DS1 with either KIR2DL5 and KIR2DS3 or KIR2DL5 or KIR2DS5 and either KIR2DS1, KIR1D, or KIR2DS4. Thus, individual gene content varies between as little as eight to as many as 14 KIR genes.

More recently, advances in gene sequencing technology has stimulated much interest in allele level KIR gene sequencing, and the genes also show a high degree of allelic polymorphism. As of December 2020, there were a total of 1532 alleles coding for 668 different proteins described in the Immuno-Polymorphism Database (Robinson et al. 2015). All KIR genes show a degree of allele polymorphism with the highest number of alleles having been described in -3DL1 (183), -3DL2 (165) and -3DL3 (228). KIR allele variation has the effect of further diversifying KIR haplotypes possessing the same set of genes (Shilling et al. 2002). A recent large study of European subjects which defined KIR genes to five-digit resolution showed that certain KIR alleles were in strong linkage disequilibrium (LD), for example KIR2DL4*00501 was in complete LD with KIR3DS1*013 and this combination was only ever found in tB01 haplotypes (Amorim et al. 2021). While KIR genes are now known to be also extremely polymorphic, there is a paucity of data associating individual KIR gene polymorphisms with disease or clinical phenotype. Allelic variation is thought to have functional consequences on the receptors generated. For example, differences in interactions have been described between KIR3DL1 and Bw4 HLA-B ligands, where the interaction of HLA-B*27:05 is weak with KIR3DL1*001 but strong with KIRDL1*002 (O’Connor et al. 2007).

### KIR ligands

HLA class I molecules act as ligands for some inhibitory KIR to provide the signal to inhibit cell activation (Colonna et al. 1993; Storkus et al. 1991; Ljunggren and Kärre 1990; Moretta et al. 1993). The HLA-C molecules as ligands for KIR can be differentiated into two groups based on the amino acid residue present at position 80. An asparagine at this position occurs in the so called C1 HLA-C, whereas a lysine occurs in the C2, and each group comprises half of all HLA-C antigens. KIR2DL2 and KIR2DL3 are highly specific for C1 molecules, whereas KIR2DL1 is specific for C2. KIR3DL1 is specific of the Bw4 epitope carrying HLA-A and -B molecules (Cella et al. 1994), which comprises an isoleucine residue at position 80, whereas an asparagine is not able to signal inhibition. KIR3DL2 binds HLA-A3 and -A11 (Dohring et al. 1996). The non-classical HLA-G has been suggested as a ligand providing inhibitory signalling to KIR2DL4 by fetal-derived trophoblast cells (Rajagopalan and Long 1999). HLA-class I molecules may also act as activating ligands albeit with less avidity. One study showed KIR2DS4 to consistently bind a subset of HLA-A and -C allotypes but with a weaker binding than that shown for KIR2DL1, 2 and 3 (Graef et al. 2009). Similarly, KIR2DS5 has been shown to be a receptor with C2-specificity at variable levels of avidity (Blokhuis et al. 2017). There are a number of reports of KIR being specific for particular peptides bound to class I HLA, for example KIR2DS1 binding HLA-C2 in the presence of specific Epstein–Barr Virus peptides (Stewart et al. 2005) and KIR2DS2 binding to HLA-A*11:01 and a vaccinia viral peptide (Liu et al. 2014). These interactions were shown to be highly dependent on polymorphic positions in the KIR receptor suggesting that allelic variation is likely to have an effect on specificity for ligand and therefore the control of NK cell effector function. The non-classical HLA-F, localized in the endoplasmic reticulum, has been shown to be a high affinity ligand for KIR3DS1 (Garcia-Beltran 2016). Non-HLA molecules have been shown to act as activating ligands, for example, heparan sulfate/heparin glycosaminoglycans as a ligand for KIR2DL4 providing an activating signal to NK cells (Brusilovsky et al. 2013).

NK cell effector function is thought to be controlled, in part, by KIR via the ‘missing self’ hypothesis (Ljunggren et al. 1990; Moretta et al. 1992). NK cells are inhibited from killing target cells by the presence of their HLA class I ligands. When these ligands are missing through downregulation such as in some viral infected or tumour cells, NK cells then kill the target cell. In the setting of transplantation, lack of the complementary KIR ligand brings about graft rejection, or in the case of hematopoietic stem cell transplant killing of residual tumour cells and recipient T cells.

NK cells undergo education to acquire functional competence by way of ‘licensing’ by self MHC molecules. This process involves MHC-specific KIR as well as other inhibitory receptors and leads to two types of self-tolerant NK cells — licensed or unlicensed (Kim et al. 2005). Interaction between KIR and their ligands is thought to be crucial for the licensing program, and NK cells licensed through two different inhibitory receptors may become more potent in their effector functions (Yokoyama and Kim 2006). NK cells are not fully reactive against MHC class I-deficient stimuli unless their inhibitory receptors have recognized self MHC class I molecules prior to this encounter (Anfossi et al. 2006).

## KIR content and transplantation

The KIR genes have been implicated in the outcome of haematopoietic stem cell transplant (HSCT). Most studies so far have focussed on gene content and the presence of certain KIR/KIR ligand mismatches and their effect on leukemic relapse. Mismatch at KIR ligands between host and donor was associated with a significantly reduced risk of relapse in haploidentical transplants in patients with acute myeloid leukaemia without leading to an increase in graft versus host disease (Ruggeri et al. 2002). Transplants using a donor with one or two KIR B haplotypes have been associated with significant improvements in overall and relapse free survival (Cooley et al. 2008). The clinical benefit of a B/x donor was not found to depend on the presence of either KIR2DL2 or KIR2DS2, or any other B-haplotype defining KIR gene. The presence of specific activating KIR genes has been associated with better HSCT outcomes. KIR2DS1 was associated with a decreased rate of AML relapse in donors with HLA-C1/C1 or C1/C2 (Venstrom et al. 2012); however, a recent large study failed to reproduce such an effect (Schetelig et al. 2020), highlighting the issue of often contradictory findings by studies, potentially caused by heterogeneity in transplant protocols, patient characteristics and disease states. Increased KIR alloreactivity has been shown to result in lower relapse and increased survival in unrelated cord blood allo HSCT by some groups and conversely with increased graft versus host disease and risk of death by others. These studies have led to the combination of KIR and HLA genotyping in the selection of stem cell donors. Discussion of the selection of unrelated HSCT donors based on HLA and KIR genotyping is outside of the scope of this article but is reviewed by Wright (2020).

Therefore, determining the presence or absence of KIR genes and KIR haplotype content has been a routine in acute myeloid leukaemia and clinical HSCT for many years; however, the role of individual KIR gene polymorphisms in HSCT and other immunopathologies has not been well studied. Recent sequencing advances, particularly with next-generation sequencing (NGS) technology, has therefore stimulated much interest in allelic level KIR sequencing and the possible association of KIR polymorphism with clinical disease and outcome studies.

## KIR genotyping

KIR genotyping methods for the last two to three decades have focussed on determining the presence or absence of KIR genes, which goes someway to determining the haplotypes present in an individual (Fig. 2). Various approaches have been applied such as polymerase chain reaction using sequence specific primers (PCR-SSP) (Uhberg et al. 1997) and PCR using sequence-specific oligonucleotide probes (SSOP) (Crum et al. 2000). Various cloning and sequencing methods have been described (Gardiner et al. 2001) to subtype KIR gene alleles; however, these are generally low throughput and time consuming. These techniques typically focus on very limited regions of sequence and as such provide very limited amounts of information about the degree and relevance of variation at the allele level. More recently next-generation (NGS) and third-generation sequencing (TGS) methods have enabled very high throughput cost effective sequencing of much larger regions — typically whole genes, haplotypes and even whole exome sequencing, which has revolutionized gene analysis. The determination of full-length sequences, including non-coding regions has provided deeper insight into gene structure as well as degree of variation, which can now be applied to functional aspects of the proteins encoded. Short-read length NGS incorporates massively parallel sequencing of clonally amplified target DNA strands to produce millions of reads for analysis; long-read TGS is able to sequence single molecules of DNA along stretches in excess of several kilobases making it highly amenable to resolving complex gene systems with long repetitive elements and structural variations (Goodwin et al. 2016). Long-read platforms may be particularly useful for discriminating the presence of fusion KIR genes created by deletions and recombination during chromosomal rearrangements. The creation of these genes may lead to the shuffling of binding and signalling domains thus altering the response of these functional distinct gene variants (Bruijnesteijn et al. 2020). Table 1 summarizes the next-generation sequencing methods discussed below. This review will now focus on these recently developed methods in their application to high-resolution KIR gene sequencing.

**Table 1:** Summary of high-resolution next-generation sequencing methods

Method	Authors	Target type	Sequencing platform	Read length	Throughput	Error rate	Cost	Reference
1	Norman et al.	KIR-specific probe capture	Illumina	Short	Medium	Low	Medium	33
2	Maniangou et al.	Long-range whole gene multiplex PCR	Illumina	Short	Medium	Low	Low	35
3	Closa et al.	Exon PCR	Illumina	Short	High	Low	Low	38
4	Wagner et al.	Exon PCR	Illumina	Short	Very high	Low	Low	39
5	Van DePasch	Whole gene PCR	Illumina	Short	Medium	Low	Medium	29
5	Roe et al.	Fosmid libraries	PacBio	Long	Low	Medium–low	Medium	36
6	Roe et al.	KIR-specific probe capture	PacBio	Long	Medium	Medium–low	Medium	37
7	Bruijnesteijin et al.	CRISPR-Cas9 enrichment	Oxford Nanopore Technology MinION	Very long	Low	Medium	Medium	40
8	Downing et al.	Long-range whole gene multiplex PCR	Oxford Nanopore Technology MinION	Very long	Medium	Medium	Low	Manuscript in preparation

### Target capture

A target capture sequencing approach has been described where libraries of sheared genomic DNA were enriched for the KIR region using a set of capture oligonucleotide probes prior to NGS using the Illumina platform (Norman et al. 2016). The KIR probes are non-overlapping 80-mers designed from a panel of reference KIR haplotypes representing 13 recognized KIR haplotypes. The probes are biotinylated and purified by binding to streptavidin-coated beads. DNA samples are sheared to 800 bp fragments and individually barcoded using Illumina dual indexes before pooling. Target capture is carried out by hybridising the pooled library to the capture probes and using streptavidin beads to separate from non-target un-hybridized DNA. This workflow was used to sequence libraries from: cell lines of the International Histocompatibility Workshop Group (IHWG); families from West Africa, 15 individuals from the KhoeSan population and exome sequences from 2112 individuals from the 1000 Genomes project. Gene content had been previously determined in the IHWG samples by PCR, and KIR alleles were previously determined in the KhoeSan samples by pyrosequencing and Sanger sequencing. An in-house bioinformatics pipeline called PING was developed which harvests KIR-specific reads for passing through modules for gene content identification, copy number determination and allele genotyping. Complete concordance was achieved with the known gene content of the IHWG samples. The analysis of 13 KIR genes in the 97 IHWG identified 144 different KIR sequences including 16 novel alleles, which were subsequently confirmed by alternative methods. KIR copy-number and allele data in the KhoeSan samples matched that previously described. From the analysis of the 1000 Genomes data, 100 novel KIR alleles were identified, which were subsequently confirmed from the genomic DNA using Sanger sequencing. One of the main benefits of the method described is the use of capture probes to obtain a sequencing target instead of PCR amplification. This abrogates PCR error and the risk of allele drop out and amplification bias. The authors state that short-read technology was chosen due to its high fidelity; however a disadvantage of short-read sequencing technology is that many of the reads would map to more than one locus due to the high degree of sequence homology in the KIR genes, these reads would then be discarded during analysis. In addition, phasing of polymorphisms could be less reliable leading to ambiguous results. The authors suggest that the method should be able to be applied to longer-read length sequencing chemistry such as Pacific Biosciences or Oxford Nanopore. This KIR genotyping workflow was recently employed to analyse a cohort of 2130 healthy individuals of European descent (Amorim et al. 2021). This study has revealed far more gene content and structure variation than any other work to date, particularly among the framework genes, with, for example, more than 6% of individuals carrying a deletion or duplication of KIR2DL4. The PING pipeline has recently been refined and updated (Marin et al. 2021), improving the throughput and enhancing copy number determination. Read misalignment with the highly homologous KIR2DS1 and KIR2DL1 genes was also improved as was the need to interpret unresolved genotypes. Even with these improvements, this study clearly highlights the difficulty in assigning reads by a bioinformatic pipeline. Misaligned reads were most frequently seen in the KIR3DP1 gene, which received reads originating from KIR3DL3, KIR3DL2, KIR2DP1 and KIR2DL4. Pairing of genes where misaligned reads were frequently seen was identified as KIR2DL1 and KIR2DS1 and KIR2DL2 and KIR2DS2. The use of short-read technology almost certainly contributed to the difficulty in aligning reads, for example, in this study, it was observed that 82% of distinct 150-mers were shared between KIR2DL5A and KIR2DL5B. It will be interesting to see this workflow applied to long-read technology.

### Long-range PCR

A long-range PCR based assay, followed by Illumina NGS, was described using six primers in a single multi-plex PCR mixture to amplify all full length KIR genes (Maniangou et al. 2017). The amplicons generated varied in size from 4 to 5 kb for KIR3DP1 to 9 to 17 kb for all other genes. Library preparation and dual indexing was performed for sequencing on a MiSeq instrument. A manual bioinformatics pipeline was employed to map sequencing reads to a reference sequence; exon sequences were extracted and compared for polymorphic bases with those contained in the IPD-KIR database. Two different bioinformatic pipelines were then employed for determining KIR allele assignment. This workflow was applied to 30 IHWG DNA samples as a validation. In all 30 samples good coverage was achieved with an average coverage of 316.55X. Allele assignment was obtained for all loci for most samples. The results were compared to those obtained by Norman’s capture-based NGS method with the finding that 100% of allele assignments at the three digit level were concordant in 11 KIR genes (*n* = 110 alleles); semi-discordance, where one allele of a heterozygous typing was different, was found in ten alleles across six loci; and fully discordant results were found in two cases. The authors state that the described method is easily implemented and possibly cheaper than a capture-based method but stressed the requirement for good quality DNA, high fidelity Taq polymerase and reliable library preparation.

### Amplicon-based exon sequencing

A different approach using PCR was described where the KIR genes were amplified to produce two amplicons, one covering exons 1 to 5 and the other covering exons 6 to 9 (Closa et al. 2018). A total of eight primers were used in a single multiplex mixture along with eight primers for class I HLA genes. The GENDX NGSgo kit was used to prepare indexed libraries for sequencing on the MiSeq. This workflow was validated with 186 DNA samples made up of 30 IHWG samples with previously determined high-resolution KIR genotypes and 156 local DNA samples previously typed for gene content by PCR-SSO. Analysis of the KIR sequences was performed by mapping reads to hg19 which were visualized with commercial bioinformatics software. KIR gene content was determined using four gene specific virtual probes per locus, which were used to find exact matches in FASTQ sequence files. KIR gene content results were found to be 97.84% concordant with the PCR-SSO result. It was estimated that around 250 samples could be analysed in a single MiSeq run using this method. Although not intended for KIR allele determination, it was suggested by the authors that this approach could be used for allele level typing when a suitable allele calling programme becomes available.

A very high throughout short amplicon PCR NGS strategy was described targeting KIR exons 3–5 and 7–9 in four separate reactions (Wagner et al. 2018). Subsequently, the reactions were pooled for each sample and index and sequencing adaptors added. Then a total of 384 samples were combined with pools of HLA, blood group and CCR5 genes for sequencing on the Illumina HiSeq2500. The inhouse bioinformatics pipeline consisted of determining gene content and copy number and then allele assignment with results given as a genotype list (GL) string. The method was validated using DNA from 93 IHWG samples and a reference set of 360 samples previously typed for KIR at the allelic level. In the IHWG samples, 413 genes were identified correctly as being absent, 224 were identified as being present but no allele called due to either a novel nucleotide or failure to meet quality criteria. Finally, 851 genes were typed at the allelic level and all but three were concordant with the previously reported result. The workflow has been used to type 1.8 million potential HSCT donors at the allelic level providing a useful insight into KIR allele frequencies. Differences were seen in the number of alleles present for each gene. Limited diversity was observed in the activating KIR genes with a single allele occurring at high frequency for some genes. Greater diversity was seen in the inhibitory KIR with evenly distributed frequencies. In a subset of 185,170 samples 5203 sequences were identified with novel positions compared to the reference database highlighting the degree to which diversity in these genes is only just beginning to be revealed. While this method demonstrates the power of high throughput NGS workflow with an impressive number of samples genotyped at very low cost, due to the incomplete coverage of the genes ambiguous results are encountered. This method has been employed recently to identify a set of 551 KIR allele group haplotypes representing 21 KIR copy number haplotypes helping to broaden the understanding of the KIR genes (Solloch et al. 2020).

### Commercial whole gene PCR

A PCR-based method was developed for commercial use by the sequencing technology company GenDX (van de Pasch et al. 2018). Nine of the 17 KIR genes were targeted in several group specific PCR mixtures which were then subjected to limited group pooling and then library preparation for Illumina MiSeq sequencing. Resulting sequencing data was analysed using the commercially available NGSEngine software. The authors noted the challenges of KIR copy number variation and high degree of homology hampering data analysis; however, the majority of samples were able to be typed unambiguously.

### Long-range sequencing using PacBIO platform

A study from the Centre for International Blood and Marrow Transplant Research (CIBMTR) looking at whole KIR haplotypes described a method using long-read technology on the PacBio platform (Roe et al. 2017). Pools of fosmid libraries targeted for KIR sequence content were constructed to span the entire KIR region in eight individuals. Full lengths of KIR locus inserts were isolated from the linearized fosmids and subjected to SMRT sequencing on the PacBio RS II. Using this approach, continuous reads of around 40 kb allowed the unambiguous assembly of two KIR haplotypes for each of the eight diploid samples. Nine distinct haplotype structures were identified in the study samples including four haplotypes not previously described. This method has recently been updated for application to population scale and clinical studies (Roe et al. 2020), replacing the technically demanding fosmid library creation with capture probe enrichment of long fragments of KIR targets which are subsequently sequenced by PacBio. Again, fully sequenced and phased diploid KIR haplotypes with fully annotated alleles were determined.

### CRISPR-Cas9 target enrichment

A CRISPR-Cas9 target enrichment method followed by Oxford Nanopore sequencing has recently been described (Bruijnesteijn et al. 2021), where guiding crRNA were designed to direct Cas9 endonuclease to cleave large overlapping DNA segments of the entire KIR region. Cleaved target sites were left with an available phosphate group which was utilized for dA-tailing and then ligation to nanopore sequencing adaptors. Nanopore sequencing relies on a signal change in the electric current passed across a synthetic nanopore as nucleotides pass through the pore. The signal produced is subsequently converted into nucleotide sequence resulting in reads potentially in excess of 100-kb long. This method was used to generate fully phased haplotypes in two subject DNAs, with a third fully phased except for one KIR gene.

### Whole KIR gene sequencing on nanopore platform

Our group is developing a full gene PCR amplicon-based method using the Oxford Nanopore Technology MinION platform. Using previously published PCR primers, all KIR genes are amplified and then subjected to a straightforward library preparation involving end repair, barcoding, pooling of around 24 samples, adaptor ligation and loading on to a MinION flow cell. The major challenge that exists for all the described methods is the accurate analysis of genes with a high degree of sequence homology, particularly where throughput gains of multiplexing and sample pooling are highly desirable. Initial analysis of the long-read length output using GENDx NGSEngine in a set of IHWG DNA samples shows promise. This method will be published separately once fully validated.

## The use of high-resolution allele level KIR typing in health and disease

### KIR in HSCT

High-resolution KIR genotyping has been employed in a growing number of studies into the allelic variation present in the KIR gene family. KIR gene allelic variation has been linked with the outcome of HSCT and solid organ transplant illustrating the need for further study of these genes at this level. Allelic variation in KIR genes allows for the characterisation of individuals by differing levels of expression, with high and low expression alleles providing stronger and weaker inhibitory signals. Donors with low inhibition KIR-KIR ligand combinations have been associated with lower relapse and higher survival, whereas combinations with higher expression, higher inhibitory activity were associated with higher relapse (Schaffer and Hsu 2016). Bari et al. have shown that allelic polymorphism in KIR2DL1 affects overall recipient survival and progression-free survival in pediatric allogeneic HSCT patients (Bari et al. 2013). Patients who received grafts with the functionally stronger KIR2DL1 allele with arginine at amino acid position 245 (KIR2DL1-R245) had better survival and lower cumulative incidence of disease progression than those patients who received a donor graft that contained only the functionally weaker KIR2DL1 allele with cysteine at the same position (KIR2DL1-C245). KIR3DL1 alleles have been segregated based on patterns of strong (*001, *002), weak (*005, *007) or lack (*004) inhibition by target cells with HLA-B subtypes and in AML patients who received HLA-compatible allografts, donor-recipient KIR3DL1/HLA-B subtype combinations that demonstrate weak or no inhibition were associated with significantly lower relapse and higher survival compared with strong inhibition combinations (Boudreau et al. 2017). A pilot study found that weak or non-inhibiting KIR3DL1 subtype donors could be identified for 93% of 211 patients who had more than one donor available, and that patients with weak or non-inhibiting KIR3DL1/HLA-B partnerships experienced higher 2-year disease free survival (Schaffer et al. 2016). Subsequently KIR allele subtyping has been shown to be feasible in prospective HSCT donor selection where KIR3DL1 alleles of potential donors were differentiated into high and low expression groups (Schaffer et al. 2021). Donors were thus assessed for KIR advantage with strength of inhibition of donor KIR3DL1 by recipient HLA-B as the priority, followed by presence of HLA-C1/KIR2DS1 and centromeric KIR haplotype content. In AML patients transplanted with donors with KIR2DL1*003 and KIR2DL3*001, seen in Cen AA individuals, a more consistent and effective NK cell response was seen which led to a significant reduction in the rate of relapse after T-replete haplo-identical HSCT (Dubreuil et al. 2020). A recent study has shown that characterising individuals for the presence of a particular B haplotype defined by allele level genotyping showed a trend to significance for protecting from relapse (Guethlein et al. 2021). In this study, allele level typing was used to differentiate Cen B/B haplotypes into Cen B01 and Cen B02, the latter of which was implicated with relapse protection.

### KIR in solid organ transplantation

The role of NK cells has been studied in the solid organ transplant setting. NK cells are known to infiltrate kidney allografts and occur at a higher frequency in the peripheral blood of patients with acute graft rejection. A role for inhibitory KIR-KIR ligand has been shown in the increased risk of chronic rejection and reduction long-term graft survival (Littera et al. 2017). KIR2DS4 and KIR2DS5 have been implicated in the outcome of renal transplantation in patients with glomerular nephritis (Nowak et al. 2012). KIR2DS4 was more frequent in patients with acute graft rejection, whereas KIR2DS5 was associated with decreased rejection. Interindividual differences in KIR and HLA class I ligand genotypes associated with differences in NK cell reactivity impact donor-specific antibody-mediated NK cell antibody dependant cell-mediated cytotoxicity in organ allografts (Rajalingam 2016). In addition, differences have been shown in the number of KIR2D expressing CD8^+^ T cells between liver transplant recipients with and without acute rejection, suggesting a role for KIR receptors in liver allograft outcomes (Lopez-Alvarez et al. 2011).

### KIR in HIV infection and control

In the context of infectious disease, KIR molecules have been shown to exhibit an effect in a variety of settings. The protection from HIV disease progression was shown to be associated with the presence of KIR3DL1 and its ligand HLA-B Bw4, particularly where the 80I is present (Martin and Gao et al. 2002). More recently, in individuals possessing HLA-B*57, a variant of the KIR3DL1 molecule has been shown to be associated with elite control of viral load and delay in disease progression (Martin and Naranbhai et al. 2018). The presence of a valine residue at position 47 of the KIR molecule was significantly higher in elite controllers of the disease. Similarly, KIR2DL3 has been shown to be protective against Hepatitis C virus whereas KIR2DL1 has been associated with recurrent human cytomegalovirus infection.

## 18th IHIWS workshop: KIR genotyping project

The 18th IHIWS Workshop taking place in Amsterdam, the Netherlands, May 2022 includes a project for sequencing full length KIR genotypes. Laboratories interested in KIR are encouraged to participate and submit samples for KIR sequencing (https://www.ihiw18.org/component-immunogenetics/kir-typing/).

The goal of this project is to characterize the nature and extent of KIR allelic diversity across human populations using next-generation sequencing (NGS). Participating labs will perform high-resolution KIR genotyping in unrelated individuals from diverse populations. Studies in families will also be conducted in order to define phased KIR haplotypes through segregation analysis.

## Conclusion

Next-generation and third-generation sequencing methods are able to generate a massive amount of sequencing data in a high throughput and cost-effective manner. The super high resolution of sequencing able to be performed will provide a detailed view of the KIR gene and haplotype structures. This knowledge can then be carried into studies of how allelic variation might affect NK cell function, outcomes in HSC and solid organ transplantation and the progression of infectious disease. Such studies are likely to provide a greater insight into how KIR molecules interact with their ligands and how variation affects affinity and specificity of the KIR molecules for their targets.
